# Impact of Ellagic Acid in Bone Formation after Tooth Extraction: An Experimental Study on Diabetic Rats

**DOI:** 10.1155/2014/908098

**Published:** 2014-11-18

**Authors:** Mazen M. Jamil Al-Obaidi, Fouad Hussain Al-Bayaty, Rami Al Batran, Jamal Hussaini, Goot Heah Khor

**Affiliations:** ^1^Centre of Studies for Periodontology, Faculty of Dentistry, Universiti Teknologi MARA, 40450 Shah Alam, Malaysia; ^2^Faculty of Medicine, Universiti Teknologi MARA, Jalan Hospital, Sungai Buloh Campus, 47000 Sungai Buloh, Selangor, Malaysia; ^3^DDH CoRe, Universiti Teknologi MARA (UiTM), 40450 Shah Alam, Selangor Darul Ehsan, Malaysia; ^4^Center of Preclinical Science, Faculty of Dentistry, Universiti Teknologi MARA, 40450 Shah Alam, Selangor Darul Ehsan, Malaysia

## Abstract

*Objectives*. To estimate the impact of ellagic acid (EA) towards healing tooth socket in diabetic animals, after tooth extraction. *Methods*. Twenty-four *Sprague Dawley* male rats weighing 250–300 g were selected for this study. All animals were intraperitoneally injected with 45 mg/kg (b.w.) of freshly prepared streptozotocin (STZ), to induce diabetic mellitus. Then, the animals were anesthetized, and the upper left central incisor was extracted and the whole extracted sockets were filled with Rosuvastatin (RSV). The rats were separated into three groups, comprising 8 rats each. The first group was considered as normal control group and orally treated with normal saline. The second group was regarded as diabetic control group and orally treated with normal saline, whereas the third group comprised diabetic rats, administrated with EA (50 mg/kg) orally. The maxilla tissue stained by eosin and hematoxylin (H&E) was used for histological examinations and immunohistochemical technique. Fibroblast growth factor (FGF-2) and alkaline phosphatase (ALP) were used to evaluate the healing process in the extracted tooth socket by immunohistochemistry test. *Results*. The reactions of immunohistochemistry for FGF-2 and ALP presented stronger expression, predominantly in EA treated diabetic rat, than the untreated diabetic rat. *Conclusion*. These findings suggest that the administration of EA combined with RSV may have accelerated the healing process of the tooth socket of diabetic rats, after tooth extraction.

## 1. Introduction

Extraction of tooth is a very prevalent dental care process. Generally, healing of socket after tooth extraction is quite a normal process; however in some cases, such as diabetes, patients might develop alveolar osteitis and hence experience late/impaired healing [1]. Therefore, it is essential to promote healing for the purpose of preventing and treating these problems. Furthermore, treatment of tooth extracted socket consequently depreciates the dimension of alveolar ridge [2]. The depreciation of the dimension has a detrimental influence on consequent implant therapy. Therefore, implant dentistry has extensively focused on the preservation of the alveolar ridge during healing.

Numerous studies have shown a link among diabetes and the increased risk for gingivitis, periodontitis, and loss of tooth attachment [3, 4]. Moreover, in case of diabetic patients, the formation of bone will be affected by hyperglycemia [5]. Additionally, diabetic patients might experience delayed healing of tooth extracted socket, as well as suffer from infections [6].

Phenolic compounds are considered as the major bioactive compounds, responsible for impacting bone health [7]. Furthermore, ellagic acid (EA), a member of flavanoids (C_14_H_6_O_8_; MW: 302.202; 3,7,8-tetrahydroxy[1]-benzopyrano[5,4,3-cde][1]benzopyran-5,10-dione), is usually produced by plants and formed as tannin, known as ellagitannins. The EA contains two lactone groups and four hydroxyl groups, in which the hydroxyl group is known to increase antioxidant activity in lipid peroxidation and protect cells from oxidative damage [8]. Currently, EA has received particular attention because of its wide array of biological properties, such as antioxidant activity, chemopreventive [9, 10] and antiapoptotic [11], antimutagenetic [12], antifibrosis [13], anti-inflammatory [14], antiatherosclerotic [15], antibacterial [16], and anti-HIV replication [17] properties. Our previous report has proved that the EA accelerated bone formation after tooth extraction in normal rats [18].

The activation of bone morphogenetic protein (BMP)-2 production by statin basically increases the formation of bone [19–21], which might induce the recovery and development of periodontal bone and ligament [22–24]. Furthermore, by means of minimizing the receptor activator expression of nuclear factor kappa-B ligand (RANKL) and cathepsin K, statins decrease resorption of bone [25]; the mix of osteoclast precursors as well as the hindrance of the osteoclasts acting ring minimizes several active osteoclasts [26].

Alkaline phosphatase (ALP) influences the maintenance of pyrophosphate to phosphate ratio for bone mineralization. ALP is considered as exceptional biochemical marker of the process of bone formation [27]. Furthermore, fibroblast growth factor-2 (FGF-2) has been considered as effective indicator of growth factor, which potentially impacts the repairing and regrowth of tissues [28, 29]. This present study is aimed at evaluating the healing of tooth extraction in diabetic rats with the expression of ALP and FGF-2, through immunohistochemical studies and histological observations. Consequently, this present study has examined the theory that formation of new bone in tooth extracted sockets is triggered by EA adjunct with RSV, by partially controlling the ALP and FGF-2 expressions in diabetic rats, as opposed to normal and diabetic control rat.

## 2. Material and Methods

### 2.1. The Animals

The experiments in this study have been conducted with the help of twenty-four male* Sprague Dawley* (SD) rats, weighing 250 to 300 g. The selected animals were bred in Universiti Teknologi MARA (UiTM) of Animal Centre and were kept in 22°C room temperature, 40% humidity, and 12 hours of daylight cycle. The selected rats were fed* ad libitum*, in accordance with standard laboratory diet and water. This study has meticulously adhered to the guidelines of the University for Animal Research (600-FF (PT.5/2)), in terms of conducting the experiments.

### 2.2. Induction of Diabetes Mellitus by Streptozotocin (STZ)

Rats belonging to one of the diabetic groups were induced with diabetes mellitus, by intraperitoneally injecting freshly prepared streptozotocin (STZ) (45 mg/kg body weight) in citrate buffer 4.5 pH. After three days, the level of blood glucose from the tail vein was determined, which was found to be >16.7 mmol/L; this indicates that the rats have been induced with diabetes mellitus [30, 31].

### 2.3. Tooth Extraction Protocol

For the experimental purpose, the right maxillary incisor of the rats was extracted by means of pliers. Prior to the extraction, the external surfaces of the pliers were ground to increase the indentation of their internal surfaces, which facilitated strongly gripping incisor of the rat during the extraction process as described by [32]. Prior to the extraction process the rats were anesthetised by intraperitoneally injecting sodium pentobarbital (50 mg/kg body weight). The rats were locally infiltrated with a few drops of 2% lidocaine containing 1 : 100,000 epinephrine, delivered into the lingual fold [33], to produce local anaesthesia and haemostasis [34]. After the tooth was extracted, the socket of all the rats was filled with Rosuvastatin (RSV). The sockets were sutured with silk thread (Ethicon 7.0, Johnson and Johnson). After surgery, each animal was subcutaneously administered with trimethoprim-sulfa (Sigma Aldrich) 15–30 mg/kg as antibiotic, every 12 h for three days [35]. During two postoperative days, animals were fed ground rat chow to facilitate feeding (to minimize both, trauma at surgical site and delay the healing process) [18, 36, 37]. After this period, standard lab block food was reintroduced. The animals were randomly divided into three groups, such as normal control group (RSV+ normal saline) and two diabetic experimental groups (RSV+ normal saline) and (RSV+EA). The normal control and diabetic control groups were orally fed with normal saline, once a day (5 mL/kg/d), and treated group was orally administrated with EA (50 mg/kg), once daily. After the surgical procedure, the health status and healing process of the sockets were periodically monitored. After the operation, four rats were sacrificed on the 14th day, and the remaining was sacrificed on the 28th day [18, 36, 38], by intraperitoneally injecting sodium pentobarbital (50 mg/kg body weight).

### 2.4. Drug Treatment

EA powder (E2250 Sigma Aldrich, USA) 12.5 mg was dissolved in 1 mL distilled water and the volume of 10 mL/kg was given to rat [39]. For this purpose 95% pure EA was used. EA was orally administered to male rats for 14–28 days, by means of a gastric tube (50 mg/kg body weight/day) [18, 36]. The rosuvastatin (crestor) was purchased from (Sigma-Aldrich. USA), it was filled into the rat socket with 10 mg/kg [20].

### 2.5. Determining Glucose Level

The levels of blood glucose were measured on a weekly basis, by snipping the tails of fasting animals based on glucose oxidase-peroxidase enzymatic technique, making use of a standard glucometer (Accu-Check Performa, Roche Diagnostic Germany).

### 2.6. Blood Collection

Upon sacrificing, the blood sample of animals was collected into heparinised tubes and centrifuged at 4000 r.p.m for 10 minutes; then the serum was collected and stored at −80°C, until the proinflammatory assays analysis was conducted.

### 2.7. Tissue Homogenization

The dissected gingival maxilla tissues were collected and cleaned four times with ice-cold normal saline, instantly inserted in normal cold saline, and homogenized at −4°C. Later the homogenate was centrifuged at 4000 r.p.m for 5 minutes. The acquired supernatant was stored in refrigerator (at −80°C) for antioxidants investigation.

### 2.8. Measurement of Protein level

The concentration protein in the tissue was determined by the Bradford dye-binding assay (Cayman, USA).

### 2.9. Proinflammatory Activity ELISA Assay

Proinflammatory parameters TNF-*α* and IL-6 were estimated in rat-serum, based on enzyme-linked immunosorbent assays (ELISA) kits, in accordance with manufacturer's instructions (Cayman. USA).

### 2.10. Determination of Antioxidant Parameters

#### 2.10.1. Lipid Peroxidation Activity in Gingival Maxilla Tissue

The thiobarbituric acid reactive substances (TBARS) level in the gingival tissue had been gauged to represent lipid peroxidation [40]. Subsequently, 250 mL of tissue homogenates was incubated in a water bath at 37°C for 60 minutes. Then, 400 mL of 35% perchloric acid was added and centrifuged at 12.000 ×g for 10 minutes. Next, 400 mL of 0.6% thiobarbituric acid solution was added to the supernatant solution, and the mixtures were inserted in a water bath and heated up for 30 minutes at 95–100°C. Then the mixtures were cooled, and the absorbance was gauged with a microplate reader at a wavelength of 532 nm. The standard curve was set up with different levels of malondialdehyde (MDA) with identical conditions.

#### 2.10.2. Activity of Superoxide Dismutase (SOD) in Gingival Maxilla Tissues

Photochemical inhibition of nitroblue-tetrazolium (NBT) was used to measure SOD activity, by measuring enzyme capacity based on [41]. For the purpose of decreasing NBT, O_2_^−^ was utilized as the basis of superoxide dismutase assays, which minimizes the production of formazan by NBT that is absorbed at 560 nm. Briefly, 20 mL of phosphate buffer or supernatants were added to test tubes, containing 1 mL of the reaction mixture (phosphate buffer 50 mM, EDTA 100 nM, and L-methionine 19.5 mM pH 7.8) in a dark room. Then, 150 mL and 750 mM of NBT and 300 mL and 10 mM of riboflavin were added; after shaking, the tubes were exposed to light (20 W) for 15 minutes. The absorbance was measured at 560 nm. The results were expressed in enzyme units, representing the amount of SOD, which is necessary to inhibit the reduction of NBT by 50%. The enzymatic activity was expressed as U/mg of gingival tissue.

#### 2.10.3. Catalase (CAT) Measurement

CAT activity was examined by the approach proposed by [42]. The enzymatic reaction was initiated by adding 20 *μ*L aliquot to the homogenized tissue and 0.5 M substrate (H_2_O_2_) in a medium, which comprised 100 mM phosphate buffer and pH 7.4. In addition, the variations in absorbance were recorded at 240 nm, and the CAT activity was calculated, with regard to nmol H_2_O_2_ consumed/min/mg of protein.

### 2.11. Histological Studies

The central maxillary incisor samples were extracted from the rats and postfixed in the 4% paraformaldehyde solution (pH 7.4) for 24 hours at 4°C. Then, the tooth and gingival samples were decalcified further with 10% tetrasodium-EDTA aqueous solution (pH 7.4) for 2 weeks at 4°C. The tissue blocks were removed and embedded in paraffin; furthermore, longitudinal serial cross sections of 5 *μ*m tissue blocks were stained with hematoxylin and eosin (Sigma Aldrich) for histopathologic examinations, and immunohistochemistry assay as described below. The results were qualitatively described in areas of newly formed bone tissue.

### 2.12. Immunohistochemistry

Next, 5 *μ*m sections of tissue blocks were placed on poly-L-lysine coated slides for the purpose of performing immunohistochemistry. The slide sections were immersed in target retrieval solution (DAKO Lot 10069393) and heated in microwave oven at 98°C for 20 minutes (maximum power 700 W) and then cooled at room temperature; anti-ALP (ab95462) and anti-FGF-2 (ab106245) primary antibodies were used. The sections were incubated with each primary antibody as mentioned above for 1 h; after rinsing thrice in DAKO wash buffer (TBS), the sections were then incubated with biotinylated secondary antibody using kit (LSAB system 2-HRP) (Lot 10069908) for 1 hour at room temperature. Following TBS rinses, the sections were incubated with streptavidin-horseradish peroxidase conjugate for additional 30 minutes at room temperature (DAKO), followed by a course of incubation diaminobenzidine (DAB) DAKO (Lot 10067468). Control immunohistochemistry reactions were performed to evaluate the specificity of the labels, omitting the primary antibody. Staining with hematoxylin was performed and used as a reference of the cytoarchitecture of the tissue.

### 2.13. Histomorphometric Assessment

The morphometric investigation of positive cells was accomplished with two slices from each rat, which were stained with immunolabeling. It is noteworthy that the information about the slices, such as to which group they belong, was not revealed to the researcher. The stained sections were analysed with the help of light microscope with 20x objective lenses, and images were captured with a digital camera (Sony Cyber-Shot DSC-W170; 10.1), installed on the microscope. The positive cell numbers or positive signal areas were determined per one slide in a total of 10 sections, mounted on the microscope, and was analyzed by (Adobe Systems Inc., San Jose, CA, USA), which is an image-processing software, based on the 10-step model, as described by Bijron et al. [43].

### 2.14. Statistical Analysis

One-way ANOVA tests were conducted to compare the effect of different experimental groups. SPSS 20 was used to perform the statistical analysis.

## 3. Results

### 3.1. Glucose Level Measurement

Table 1 illustrates that both diabetic groups as compared with normal control group (*P* < 0.05). While, untreated diabetic rat displayed significant increased in glucose level when compared with EA treated group (*P* < 0.05). EA treated diabetic group has significantly decreased blood glucose levels (*P* < 0.05) after one week following EA treatment.

### 3.2. Impact of EA on Proinflammatory Parameters

**Figure 1:**
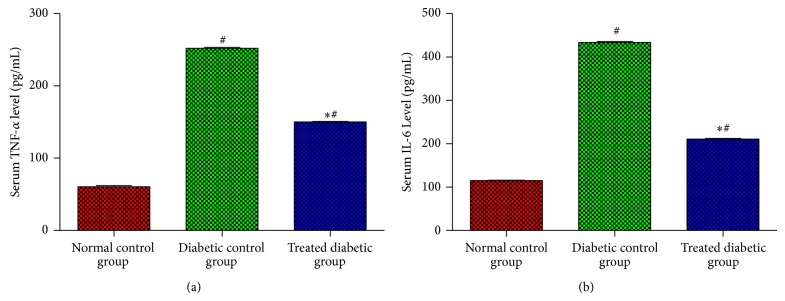
Effects of EA on inflammatory cytokines TNF-*α* (a) and IL-6 (b) levels in serum experimental rats after tooth extraction. Each bar represents the mean ± SD. ^*^Significant difference compared to diabetic control group (*P* < 0.05). ^#^Significant difference compared to normal control group (*P* < 0.05).

After tooth extraction, the levels of inflammatory cytokine in the serum of the normal and diabetic rats were determined.  Figure 1 illustrates that level of inflammatory cytokine was significantly lower in normal control group, as compared with both diabetic groups (*P* < 0.05). However, the levels of TNF-*α* and IL-6 in the diabetic control group were significantly increased, as opposed to the diabetic rat treated with EA (*P* < 0.05), which prevented the release of those cytokines, after tooth extraction.

### 3.3. Impact of EA on Antioxidant Activities

**Figure 2:**
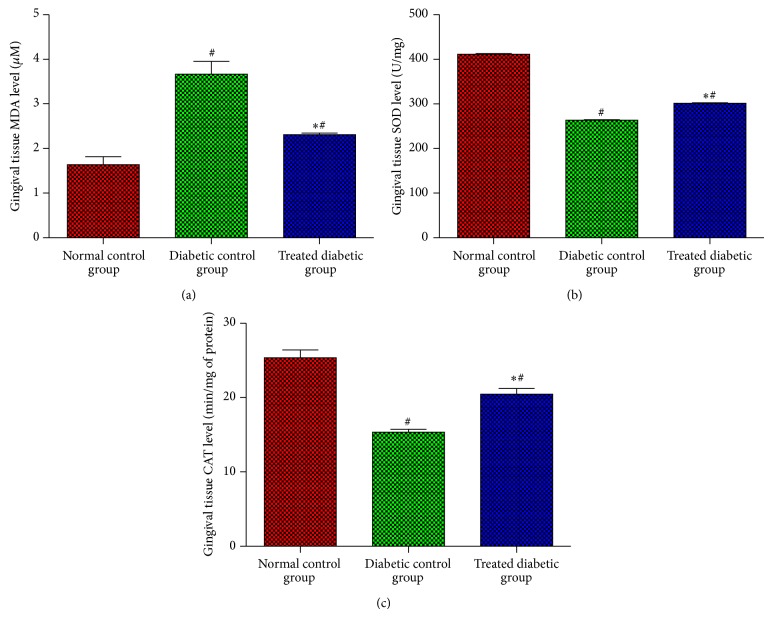
Effects of EA on antioxidant status activities MDA (a), SOD (b), and CAT (c) levels in gingival tissue after tooth extraction in normal and diabetic rats. Each bar has been represented as mean ± SD. ^*^Significant difference compared to diabetic control group (*P* < 0.05). ^#^Significant difference compared to normal control group (*P* < 0.05).

Antioxidant activity plays a key role in accelerating healing process; this study has suggested that administration of EA might potentially impact the production of antioxidant parameters, such as CAT and SOD, and inhibits the effects on lipid peroxidation parameter MDA.  Figure 2 illustrates the effects of EA in homogenized tissue of normal and diabetic rats after tooth extraction. The results of this study have indicated that level of MDA was significantly decreased in normal control group as against both diabetic groups (*P* < 0.05); on the other hand, diabetic rats, treated with EA, have revealed decreased MDA levels in homogenized tissues (*P* < 0.05), compared with untreated diabetic control group. Furthermore, normal control group has presented higher SOD and CAT levels, as compared to both the diabetic groups (*P* < 0.05). However, diabetic rats, treated with EA, have showed significant increase in CAT and SOD levels, compared with untreated diabetic group (*P* < 0.05).

### 3.4. Histological Observations

#### 3.4.1. Effect of EA on Alveolar Bone at 14 and 28 Days

**Figure 3:**
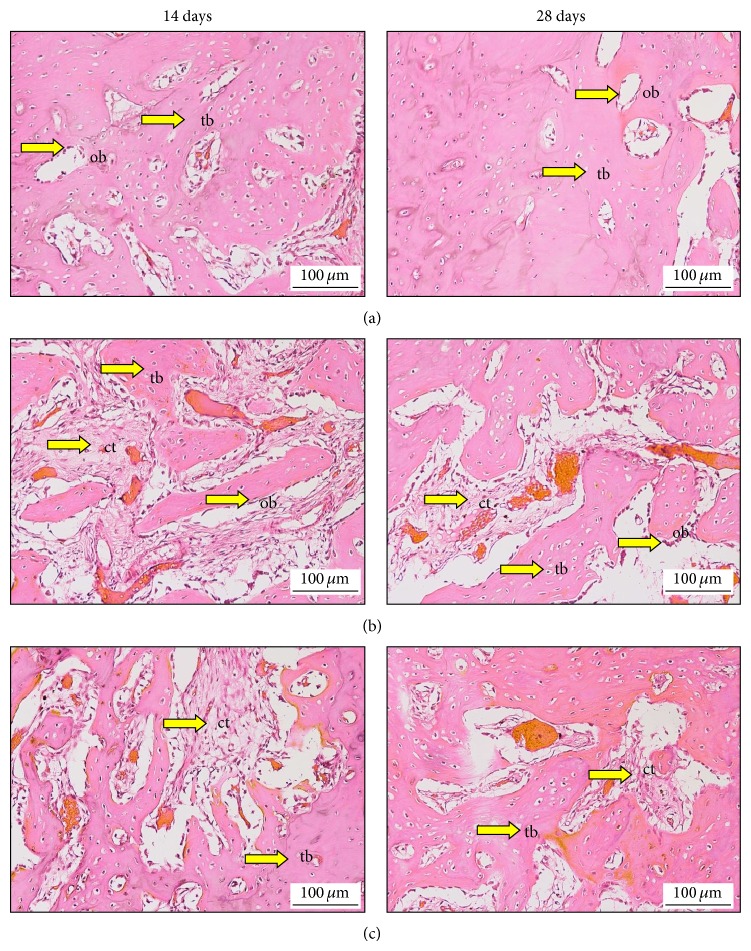
The healing process in the alveolar bone in normal and diabetic rats at 14 days (left side) and 28 days (right side). (a) Normal control group treated with normal saline (5 mL/kg). (b) The untreated diabetic rat was fed with normal saline (5 mL/kg), and (c) treated diabetic rat administrated with EA (50 mg/kg/b.w.). Trabeculae bone (tb), connective tissue (ct), osteoblasts (ob). Histological sections stained with haematoxylin and eosin 200x.

Figure 3 illustrates the left side alveolar bone section of normal, untreated, and EA treated diabetic rats; the alveolar bone section was stained with hematoxylin and eosin for 14 days after tooth extraction. Panel (a) indicates that the bone formation has been remarkably increased. Panel (b) of untreated diabetic rat reveals insufficient connective tissue (ct), thin collagen fibres, and incomplete or abortive attempts for bone formation (osteogenesis), with prominent blood vasculatures and RBCs extravasations and a smaller amount of osteoblasts (ob). Panel (c) of diabetic animals treated with EA reveals receding in the connective tissue (ct) of newly formed vascular structures, with a marked increase in the coarse meshwork formation (intramembranous ossification), and it is also evident that the trabeculae bone is well organized with obvious higher amount of osteoblast (ob).

Figure 3 illustrates right side alveolar bone section of normal control, untreated diabetic and EA treated diabetic rats, stained with hematoxylin and eosin (see Section 2) for 28 days, after tooth extraction. Panel (a) shows a significant increase in the bone formation. Panel (b) of diabetic untreated rats reveals receding of the connective tissue (CT) in newly formed vascular structures, with a significant increase in the coarse meshwork formation (intramembranous ossification) with less osteoblast (ob), and indicates the presence of secondary osteons in primitive trabecular bone; furthermore, the trabecular bone is also not properly organized. Panel (c) of diabetic animals treated with EA reveals mature trabecular bone formation and shows enhancement of trabecular bone formation and it is also well organized, with lesser connective tissue (ct).

### 3.5. Immunohistochemistry Observations

#### 3.5.1. Effect of EA on ALP Activity at 14 and 28 Days

**Figure 4:**
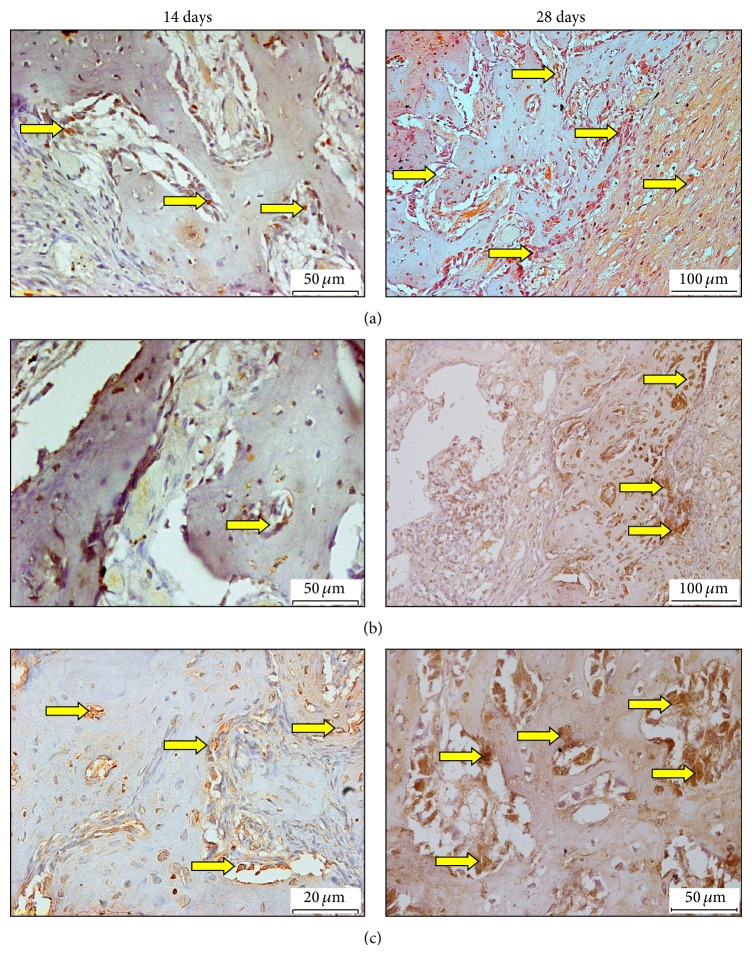
Expressions of ALP immunolabeling in socket normal and diabetic animals at 14 (left side) and 28 (right side) days after tooth extraction of the right maxillary incisor. (a) Normal control group, (b) diabetic untreated rat (RSV+ normal saline), (c) diabetic rat treated with (RSV+EA). DAB with hematoxylin counterstaining, original 20x (ALP = arrows; DAB = diaminobenzidine).

Immunoreactivities of ALP of normal and diabetic rats were assessed on the 14th and 28th days, after tooth extraction (Figure 4). The analysis has revealed that, 14 days after tooth extraction, the normal control group has shown positive labelling, as compared with both diabetic groups. However, the results have presented obvious intensive staining of ALP in fibroblast and osteoblast, similar to cells in group treated with EA, as against the diabetic control group, which has showed faint immunostaining reactivity. Similarly, the level of ALP expression was strong in normal control group, as against both diabetic groups on 28th day. Furthermore, diabetic rat treated with EA has revealed higher ALP expression than untreated diabetic rat, which displayed weak immune-labelling effect.

#### 3.5.2. Effect of EA on FGF-2 Activity at 14 and 28 Days

**Figure 5:**
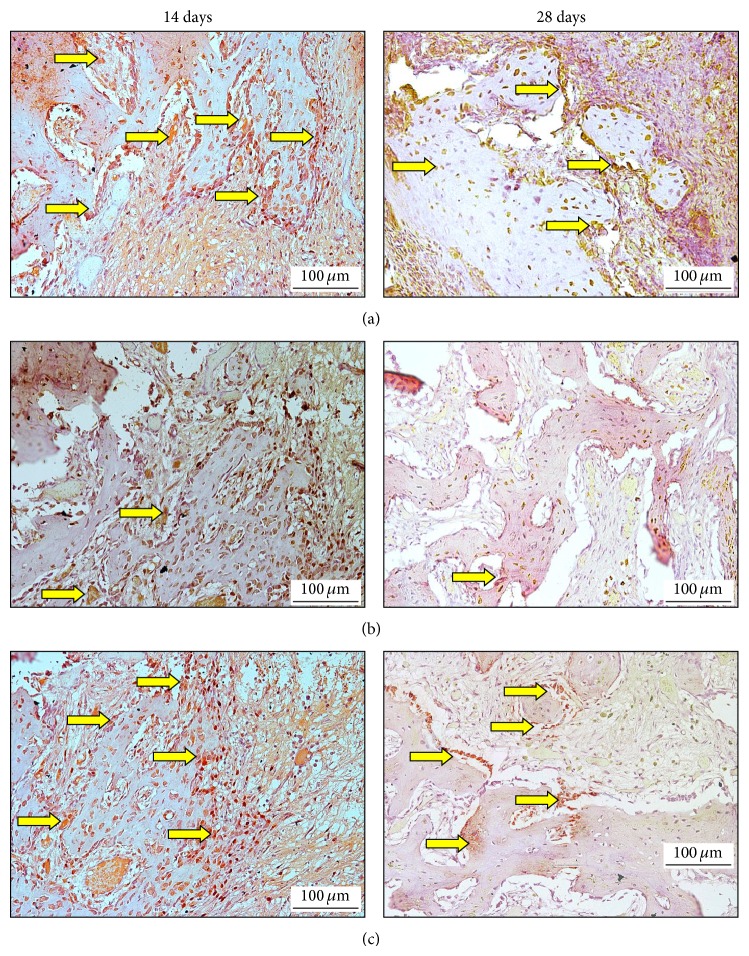
Expressions of FGF-2 immunolabeling in socket normal and diabetic animals at 14 (left side) and 28 (right side) days after tooth extraction of the right maxillary incisor. (a) Normal control group, (b) diabetic untreated rat (RSV+ normal saline), (c) diabetic rat treated with (RSV+EA). DAB with hematoxylin counterstaining, original 20x (FGF-2 = arrows; DAB = diaminobenzidine).

Immunoreactivities of ALP of normal and diabetic rats were assessed on the 14th and 28th days, after tooth extraction (Figure 5). The analysis has revealed that, 14 days after tooth extraction, the normal control group has revealed obvious FGF-2 expression level as against both the diabetic groups. However, during this period, immunostaining for FGF-2 was observed in extracellular matrix and bone spicules, and FGF-2 was very intense in diabetic rats treated with EA; nevertheless, diabetic rats, which received normal saline only, have presented decreased FGF-2 labelling. Similarly, after 28 days, the diabetic rats treated with EA have showed positive immune-labelling of FGF-2, as opposed to the group that received normal saline.

### 3.6. Histomorphometric Analysis

**Figure 6:**
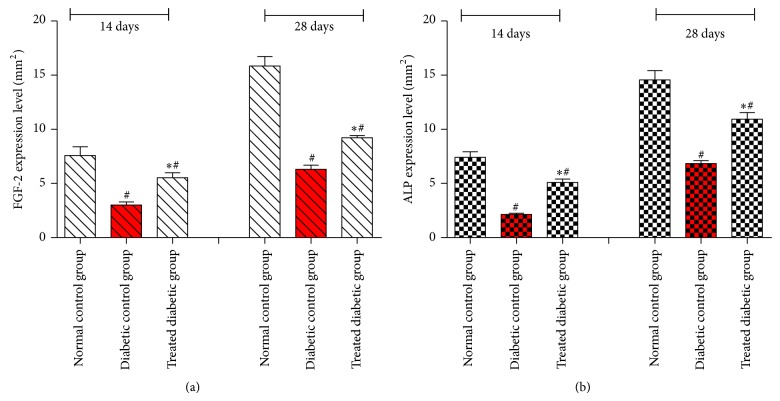
Quantitative analysis of FGF-2 (a) and ALP (b) expression levels on 14th and 28th days in socket of experimental groups. Data were quantified by image analysis software as mean ± SD and analyzed by one-way ANOVA (SPSS version 20); (∗) indicates significant difference *P* < 0.05 versus untreated group. (#) indicates significant difference *P* < 0.05 versus normal control group.

Figure 6 illustrates FGF-2 and ALP immunoreactivity in normal and diabetic rats on 14th and 28 days. The histomorphometric analysis has revealed FGF-2 immunoreactivity in the socket of diabetic rats. The normal control group has presented significant increase in FGF-2 expression level, when compared to both diabetic rats (*P* < 0.05) on 14th and 28th days. However, the socket of diabetic rat, treated with EA, has exhibited significant increase in FGF-2 expression level, when compared with untreated diabetic rat (*P* < 0.05) after 14 and 28 days of tooth extraction. Meanwhile, ALP expression level was significantly higher in normal control group (*P* < 0.05) when compared to both diabetic rats. Nevertheless, ALP expression level was significantly enhanced in diabetic group treated with EA, when compared with untreated diabetic group (*P* < 0.05) after 14 and 28 days.

## 4. Discussion

This research has investigated the antioxidants and anti-inflammatory properties of EA and RSV in terms of alveolar bone healing after tooth extraction, in diabetic rats. Proinflammatory cytokines, such as TNF-*α* and IL-6, were presented by activating macrophages, lymphocytes, and endothelial cells [44]. Discharge of proinflammatory cytokines triggers changes in the tissues, called inflammation [45]. In addition, TNF-*α* plays a central role in pathophysiology of bone [46]. This study has identified significant decrease in TNF-*α* and IL-6 in normal control group, as against both diabetic groups. However, the cytokines were significantly increased in the serum of untreated diabetic rats, as opposed to the diabetic rats treated with EA; this finding is in line with [47], which has reported that EA has significantly attenuated the proinflammatory cytokines in normal control group, compared with diabetic rats. A number of studies have proved that the separation of osteoblast and formation of mineralized nodule* in vitro* are suppressed by TNF-*α* [46, 48, 49]. Furthermore, TNF-*α* stimulates several signalling pathways in osteoblasts, such as p38 mitogen-activated protein kinases (MAPK) [50], Jun N-terminal kinases (JNK) [51], nuclear factor kappa-light-chain-enhancer of activated B cells (NF-*κ*B) [52], and Smurf1 [53], and increases apoptosis [46]. Moreover, metabolic diseases, such as diabetes, also show higher TNF-*α* levels in the blood serum [54]. In addition, the level of IL-6 in the serum changed same as the TNF-*α* level [55]. Several lines of evidence indicate that activation of NF-*κ*B can be controlled by ROS; and antioxidants block proinflammatory cytokine transcription, by preventing the migration of NF-*κ*B to the nucleus [56, 57]. Numerous studies have reported antioxidative properties of EA against oxidative stress in PC 12 cells [58]. Precisely, EA has been proved effective in preventing the activation stimulated by IL-6 and TNF-*α* of activator P1 and MAPK, in activated pancreatic stellate cells* in vitro* [59].

Furthermore, EA also possess anti-inflammatory capabilities, which downregulates inducible nitric oxide synthase (iNOS), cyclooxygenase (COX-2), TNF-*α*, and IL-6, by suppressing NF-*κ*B carcinogenesis in rats [60]. In addition, it has been proved that EA is capable of protecting the kidneys of diabetic rats against glycative and inflammation development, by minimizing the development of glycative biomarkers, such as pentosidine, controlling the aldose reductase activity, and minimizing the discharge of inflammatory cytokines, such as IL-1*β* and IL-6 [61]. Therefore, EA is capable of reducing the activity of proinflammatory cytokines in socket of diabetic rat. This study has showed that proinflammatory cytokines are capable of inhibiting bone formation/osteogenesis* in vivo*. However, the EA compound has retrieved the proinflammatory cytokines after tooth extraction in diabetic rat.

The results of this study have also revealed that normal control group has displayed significant decrease in MDA level and significant increase in SOD and CAT, when compared with both diabetic rats; this finding has also been confirmed by [47]. On the other hand, untreated diabetic rat has expressed significantly higher level of MDA maxilla tissue, compared with EA treated diabetic rat after tooth extraction. However, antioxidant parameters SOD and CAT are significantly increased in diabetic rat treated with EA, as opposed to untreated diabetic group. According to [62], production of free radicals after tooth extraction is increased by a number of phagocytic cells, including monocytes, macrophages, and neutrophils, due to formation of oxygen-derived free radicals, where those cells gather on nearby bone surfaces.

Increased levels of ROS lead to oxidative stress and damage critical biomolecules, resulting in deleterious biological effects. Malondialdehyde (MDA) is one of the end products with low-molecular-weight; increased MDA levels can be an indicator of oxidative stress. EA is a naturally occurring plant polyphenol [63] that reveals antioxidative properties both* in vivo* [64] and* in vitro* [65]. These levels of MDA reduced by EA likely demonstrate that it might be a novel agent to protect the tissues from oxidative stress after tooth extraction [66]. Consistent with our results, some studies have identified that EA may exert a potent scavenging action on bot O_2_^−^ and OH^−^, as well as lipid peroxidation [18, 36, 67].

Furthermore, Uzar et al. [68] have demonstrated that EA has the ability to reduce the oxidative damage of sciatic and brain tissues in diabetic rat. Moreover, in our previous research [18, 36] we had reported that EA attenuated the MDA level in normal tooth extracted rat. Meanwhile, cells possess various antioxidant protection features for neutralizing the detrimental consequences of ROS [69]. Superoxide dismutase (SOD) is one of the antioxidant enzymes that defends the cells from the terrible impact of ROS, to make sure that O_2_ is effectively modified as H_2_O_2_ [70, 71]. A significant reduction of SOD activity was found in gingival tissue, adjacent to deep periodontal pockets (Ellis et al. 1998), which agrees with this present study. EA is a flavonoid, which acts as antioxidant and free radical scavenger [8, 10, 68, 72], which is one of the reasons for the reduced level of SOD in gingival tissue.

In parallel with our recent research a number of other studies have revealed that EA has the ability to enhance the SOD level in maxilla tissues after tooth extraction in rats. Similarly, CAT is one of the antioxidant enzymes that detoxify hydrogen peroxide. Suda et al. [73] have demonstrated that CAT is capable of suppressing osteoclastic differentiation* in vitro*. Additionally, in case of postmenopausal women with osteoporosis, CAT activity was found to be lowered [74, 75]. Accordingly, the treatment of ovariectomised mice with CAT inhibits bone loss [75], suggesting a crucial role of CAT in alveolar bone formation after tooth extraction. Furthermore, SOD radicals form hydrogen peroxide, which in turn is decomposed as water and oxygen by CAT, thereby preventing the formation of hydroxyl radicals [76]. According to Al Batran, Al-Bayaty, and Al-Obaidi (2013) who reported that the andrographolide compound has antioxidant property, which prevents alveolar bone resorption, after measuring glutathione (GSH) by morphometric method it has been identified that bone resorption was induced by* Porphyromonas gingivalis* in rats. Several studies have reported that EA is capable of attenuating the effect of oxidative stress on the tissue, via CAT stimulation in diabetic animals [77, 78].

In terms of bone formation, mature osteoblasts synthesize bone matrix proteins, including bone-specific ALP [79]. Therefore, ALP is assessed as marker of bone formation. Our results have displayed that ALP immunolabeling has been clearly developed in normal control group, against both diabetic rats. Nevertheless, ALP immunolabeling has been markedly decreased in the socket of untreated diabetic rat against socket of diabetic rats treated with EA, as it displayed obviously higher ALP expression. These results were also confirmed by histomorphometric analysis. Ellagic acid was the main phenolic compound in the berries [80]. Consistent with our project, [81] has showed that blueberry has increased mRNA levels of ALP by 75%, in rats with defective bones. In addition, [82] has found that the serum activity of ALP was increased in groups treated with flavonoids, probably due to the decreased bone resorption. In preosteoblastic and osteoblast-like cell lines, vitamin C promotes collagen synthesis, vitamin D-stimulated expression of ALP, and mineralization [83]. Additionally, Prior et al. [84] have identified that EA has the highest antioxidant capacity, as against other compounds, such as vitamin C. Additionally, [85] has reported that oxidative stress affects the mineralization and downregulates the osteogenic marker of ALP in MC3T3-E1 cells, treated with H_2_O_2_. In the present study, the diabetic rats have showed delayed bone healing after tooth extraction due to decreasing ALP level. Moreover, in case of diabetic rats, level of serum ALP was significantly lower than those of the normal control rats, suggesting reduced bone formation [86].

Fibroblast growth factor-2 (FGF-2) is one of the proteins that could induce vascularization. This protein could also improve proliferation and adhesion properties of the mesenchymal stem cell (MCSs) and have protective effects on these cells (Kollar et al. 2009). However, the role of FGF in fracture healing is not well understood, because FGF-2 not only induces angiogenesis [87–89]. Our results have revealed that the FGF-2 immunolabeling was markedly increased in normal control group, against both diabetic rats. However, FGF-2 immunolabeling was decreased in untreated diabetic rat, as opposed to diabetic rat treated with EA, after tooth extraction, which is also confirmed by histomorphometric analysis. Earlier* in vivo* studies have shown that FGF-2 potentially induces bone regeneration [90]. Similarly few other studies have revealed that FGF-2 accelerates the mineralization of osteoblasts and MSCs [91, 92].

Under diabetic conditions, the production of endogenous bFGF mRNA is reduced [93] which may reduce the ability of bFGF to promote mitosis and control proinflammatory signalling cascades, which affect apoptosis [94].

The speculation of the increase of FGF-2 in group treated with EA is attributed to the induction of osteogenic differentiation by FGF-2, through Runt-related transcription factor-2 (Runx2) activation in vascular smooth muscle cells [95]. Recently, it was shown that stimulation of osteoblast differentiation and bone formation by FGF-2 is mediated by modulation of the Wnt signalling pathways (Wnt) [96]. The canonical Wnt/*β*-catenin signalling is another key pathway for regulating bone formation and remodelling and contributes to osteoblastic differentiation [97]. Additionally, [98] has found that* Herba Epimedii* contains EA, which enhances the WNT pathway signalling in bone formation experiment. Therefore, it is postulated that the Wnt/*β*-catenin signalling may be involved in EA enhanced osteogenesis. In our previous research we have identified that alveolar bone healing was enhanced by EA after tooth extraction, by increased osteocalcin (OSC) and osteopontin (OPN) expression levels in normal rats [18, 36].

Furthermore, using MC3T3-E1 cell same as osteoblast precursor mode, [99] has found that pomegranate seed oil (PSO) is capable of significantly stimulating osteoblastogenesis, due to increased transcriptional levels of major osteoblast lineage markers (ALP, bone sialoprotein (BSP), OSC, OPN). Based on the increase in RunX2 expression level, it has been hypothesized that the major osteoblastic transcription factors [100] may contribute to the presently observed improved osteoblast functions. RunX2 is known as the master osteogenic transcription factor, which plays a major role in the process of osteoblast maturation [101]. Along with RunX2, involvement of the Wnt/*β*-catenin signalling pathways may also contribute to PSO effects, for the same reasons [97]. Substantial amounts of EA are found in the pomegranate seed oil (*Punica granatum*) [102].

## 5. Conclusion

This study had identified that EA is a natural compound which is effective in preventing bone loss caused by tooth extraction in diabetic rat; this impact can be attributed to the increased ALP expression level and FGF-2 as well. Proinflammatory cytokines were retrieved in the serum of diabetic rat after tooth extraction by means of EA treatment. Furthermore, antioxidant status was improved in gingival tissue of diabetic rat after being treated with EA, which reduced the MDA level as indicator for oxidative stress.

## Figures and Tables

**Table 1:** The effect of EA on fasting blood glucose level of normal and diabetic experimental rats.

Group	Fasting blood glucose level (mmol/L)				
	Pretreatment week	Treatment weeks			
	Week 0	Week 1	Week 2	Week 3	Week 4
Normal control group	6.03 ± 0.33	6.33 ± 0.44	6.01 ± 0.35	6.13 ± 0.62	6.04 ± 0.60
Untreated diabetic control	20.58 ± 1.02^#^	19.67 ± 1.04^#^	18.93 ± 0.56^#^	18.32 ± 0.74^#^	18.00 ± 0.48^#^
Treated diabetic group	19.65 ± 1.47^#^	17.23 ± 0.32^∗#^	15.88 ± 0.42^∗#^	13.85 ± 0.29^∗#^	12.36 ± 0.42^∗#^

Mean values revealed by the Tukey comparisons test (*P* < 0.05). Values denote mean ± SD. ^*^Significant difference compared to untreated diabetic control (*P* < 0.05). ^#^Significant difference compared to normal control group (*P* < 0.05).
